# LOAC2: The Improved Version of the Light Optical Aerosols Counter for Measurements at Ground Level and Within the Atmosphere Under Balloons

**DOI:** 10.3390/s26123786

**Published:** 2026-06-14

**Authors:** Jean-Baptiste Renard, Gwenaël Berthet, Matthieu Jeannot, Patrick Jacquet, Benjamin Langerome, Thomas Lecas, Stéphane Chevrier, Emmanuel Briaud, Gilles Chalumeau, Florent Grenard, Benjamin Charpentier, Maylis Gaulin, Slimane Bekki, Jérôme Giacomoni

**Affiliations:** 1 Laboratoire de Physique et Chimie de l’Environnement et de l’Espace, Centre National de la Recherche Scientifique, Université d’Orléans-CNES, 45071 Orléans, France; gwenael.berthet@cnrs-orleans.fr (G.B.); patrick.jacquet@cnrs-orleans.fr (P.J.); benjamin.langerome@cnrs-orleans.fr (B.L.); thomas.lecas@cnrs-orleans.fr (T.L.); stephane.chevrier@cnrs-orleans.fr (S.C.); emmanuel.briaud@cnrs-orleans.fr (E.B.); gilles.chalumeau@cnrs-orleans.fr (G.C.); 2 AirCityWeather, 41300 Salbris, France; matthieu.jeannot@neorion-concept.fr; 3 Institut des Sciences de la Terre d’Orléans, Centre National de la Recherche Scientifique, Université d’Orléans, 45071 Orléans, France; 4 Meteomodem, 77760 Ury, France; fgrenard@meteomodem.com (F.G.); bcharpentier@meteomodem.com (B.C.); 5 Laboratoire Atmosphères, Observations Spatiales, Institut Pierre-Simon-Laplace, Sorbonne Université, Université de Versailles Saint-Quentin-en-Yvelines, Centre National de la Recherche Scientifique, 75005 Paris, France; maylis.gaulin@latmos.ipsl.fr (M.G.); slimane.bekki@latmos.ipsl.fr (S.B.); 6 Aerophile SAS, 75015 Paris, France; giacomoni@aerophile.com

**Keywords:** optical aerosol counter, concentration, particulate matter, typology, monitoring, atmosphere, balloon

## Abstract

**Highlights:**

**What are the main findings?**

- LOAC2 light aerosols counter.
- Counting and typology of atmospheric particles between 0.15 µm and 90 µm, for up to 30 size classes.

**What are the implications of the main findings?**

- Good agreement with other instruments.
- Can be used at ground level for monitoring and field campaigns, and on all kinds of balloons, including weather balloons, up to an altitude of 35 km.

**Abstract:**

The new LOAC2 optical aerosol counter is designed to detect liquid and solid particulates across 19 to 30 size classes within the 0.15–90 µm size range, and to provide their main typology. The instrument can be used at ground level and on all kinds of balloons, including weather balloons, up to an altitude of about 35 km. The measurements are based on principles established for the previous version of LOAC, now incorporating improved electronics and detection geometry. Counting is performed at small scattering angles in the diffraction domain, making it insensitive to the refractive indices and the porosity of the particles, thus allowing a direct relationship between scattered intensity and aerosol size. Typology identification is now performed at three additional scattering angles, where the scattered flux is highly sensitive to the refractive index of the different aerosol families present in the atmosphere. The calibration was conducted using calibrated spherical and irregular grains, as well as different types of solid particles. Several intercomparison sessions with other counters and with reference mass-concentration air quality monitoring stations were carried out indoors, in an atmospheric simulation chamber, and in outdoor ambient air. The agreement between LOAC2 and the other instruments is good, confirming the ability of LOAC2 to be used for scientific studies and for monitoring atmospheric aerosols.

## 1. Introduction

Accurately measuring aerosol concentrations, compositions, and size distributions in the different parts of the atmosphere is of great interest. Depending on their origin, nature, and altitude, aerosols contribute to atmospheric chemistry, affect the atmospheric radiative budget, and have consequences for human health.

Aerosols are diverse, ranging from natural emissions of terrestrial biogenic particles, wildfire smoke, desert dusts, sea salts, volcanoes, and interplanetary materials [1,2] to anthropogenic activities such as transport, industry, agriculture, and heating [3]. These sources emit primary particulates directly into the atmosphere, but they also lead to the formation of secondary particulates through reactions between gaseous species [4]. Aerosols also participate in atmospheric chemistry through heterogeneous reactions, mainly in the troposphere, although some processes also occur in the stratosphere [5,6]. 

Depending on their altitude and composition, aerosols can have either a negative or positive effect on the greenhouse effect feedback loop [7]. Stratospheric aerosols originating from explosive eruptions (i.e., volcanoes capable of injecting aerosols or their precursors directly into the stratosphere) can induce negative radiative forcing lasting several months to years, as observed after the 1991 eruption of Mount Pinatubo [8]. In contrast, carbonaceous particles may produce either negative or positive forcing depending on their altitude and the underlying surface albedo [9], although their overall global forcing relative to pre-industrial times may be negative [10,11]. Conversely, a decrease in particulate pollution concentrations can lead to a local positive radiative forcing [12].

Particulate matter (PM) has significant effects on human health, particularly solid PM during pollution events [13]. Depending on its composition and size, with the smaller PM being the most harmful, it can contribute to cardiovascular, respiratory and neurodegenerative diseases as well as cancers [14]. In particular, its role in exacerbating COVID-19 mortality peaks has been recently highlighted [15,16,17]. In addition, pollens (bioaerosols) are associated with the increasing prevalence of allergic diseases worldwide, a trend that may also be linked to anthropogenic air pollution [18]. A better understanding of their vertical distribution and transport is needed to improve allergy forecasting and public health information [19].

Due to the multiplicity of their sources and their sensitivity to meteorological parameters governing transport, accumulation and dispersion, aerosols exhibit strong spatial and temporal variability throughout the atmospheric column. This variability requires a large number of measurements to accurately assess and monitor their concentrations and compositions. Such studies can be conducted using satellite instruments, which provide extensive spatial coverage but are limited in horizontal and vertical resolution [20,21,22,23,24]. In addition, estimates of size distribution and composition are indirectly derived from these remote sensing measurements [25,26,27]. Therefore, it is mandatory for satellite measurements to be complemented by in situ measurements at ground and inside the atmosphere to better characterize the physical and chemical properties of the aerosols.

All these results highlight the need to monitor aerosols concentrations over time and throughout the entire atmosphere using in situ measurements. Measuring aerosols at ground is relatively easy, as various techniques are available to detect particles ranging from a few nm to few hundreds of µm, as well as determining their size distribution, nature and composition (e.g., [28,29,30]). In contrast, measurements within the atmosphere are more challenging. Two main platforms can then be used: aircrafts and balloons. Research aircrafts can reach an altitude of up to 21 km but campaigns are costly. They can carry a wide range of complex instruments, such as aerosol counters based on different detection techniques, mass-spectrometers, and sampling devices, often along with the scientific teams operating them [31,32,33,34,35]. Balloons can reach altitudes up to 40 km, allowing for the retrieval of vertical profiles of aerosols concentrations in the troposphere and stratosphere. They can carry counting, sampling and light-scattering instruments; however, the low temperatures and low pressures encountered at high altitude inside the gondola impose strong constraints that limit the range of instrumental techniques available [36,37,38,39,40,41,42]. Large balloons capable of carrying payloads of several hundreds of kg are complex and expensive to operate. In contrast, low-cost weather balloons can reach similar altitudes and are easier to launch, but they are limited by a payload mass of only a few kg. Finally, drifting balloons operated at a constant altitude in the boundary layer and in the lower stratosphere, can remain in flight from tens of hours to several months, and can carry payloads up to few tens of kg.

A large number of campaigns have been conducted using aircrafts and balloons and have provided, for example, measurements of the size distribution within transported desertic dust and wildfire plumes, the composition of solid particles including the meteoritic material, the temporal evolution of aerosols from the Mount Pinatubo eruption in the middle stratosphere, plumes in the lower stratosphere resulting from moderate to strong volcanic eruptions, and the level of background stratospheric aerosols [31,32,33,34,35,36,37,38,39,40,41,42].

Among these instruments, the first version of Light Optical Aerosols Counter (LOAC), sold by Metemodem Company, Ury, France was developed and has been operated since 2013 to measure aerosol concentrations in 19 size classes ranging from 0.2 to 30 µm, and to provide particle typology [43]. Measurements can be conducted outdoors at ground level during field campaigns, as well as during flights under various types of balloons up to an altitude of ~35 km (tethered balloons, weather balloons, balloons, drifting balloons, and large zero pressure balloons) from different locations worldwide [44,45,46]. About 300 flights have been performed under a wide range of geophysical conditions. In some cases, flights were conducted under alert to intercept specific plumes, such as the plume from the Tonga eruption a few days after the January 2022 event [47].

LOAC is now an aging instrument with several limitations, particularly in measuring submicronic particles at low concentrations, in typology identification, and in its sensitivity of the temperature variations encountered during balloon flights. Therefore, a new version of the instrument, called LOAC2, has been developed to strongly improve the counting and identification performance for all kinds of particles present in the atmosphere. Two versions exist, a light one for use under weather balloon, and a second one with a more robust box for field campaigns.

## 2. Materials and Methods

### 2.1. Principle of Measurements

The previous and present versions of LOAC, like all optical particle counters (OPCs), are based on measurements of light scattered by individual liquid and solid particles that cross a light beam inside an optical chamber, where the scattered flux is recorded by detectors [44,48,49,50,51]. These particles are injected into the chamber using a pump. The light scattered by the particles depends on 4 parameters [52,53]: their size, their refractive index, their porosity and the scattering angle (defined as the angle between the directions of illumination and observation). Most OPCs perform measurements at a large scattering angle, typically around 90°, to limit possible stray light contamination inside the chamber. The aperture, or field of view, is often several tens of degrees in order to record as much scattered light as possible.

The instruments are calibrated using Mie scattering calculations [54] and spherical; well-calibrated, monodisperse particles made of latex, plastic or glass. The main limitation of this approach lies in the sensitivity of the scattered light to the refractive index and porosity of the particles [53]. Therefore, calibrations are adjusted by assuming mean optical properties of the particles expected to be measured, such as liquid droplets, mineral or carbonaceous particles, or average urban pollution particles.

The LOAC concept, designed to detect irregularly shaped particles, adopts another approach. Counting measurements are performed at a small scattering angle, i.e., below 20°, where diffraction dominates the scattered light intensity in the case of irregular particles [43,55]. The advantage of performing measurements at such small angles lies in the weak sensitivity of diffraction to the refractive index and the porosity of the particles. Therefore, a direct relationship between the recorded scattered light intensity and particle size can be established, without requiring assumption about the nature of the particles.

The main drawback of this approach is that the size of perfectly spherical particles, such as metal or latex beads, for which Mie scattering oscillations dominate the scattered intensity, cannot be accurately determined. Fortunately, solid atmospheric particles are not perfect spheres but are irregularly shaped [56], and their departure from sphericity and surface roughness are sufficient enough to be considered as irregular particles [55]. Even liquid droplets may not be perfectly spherical. They are in motion and can be deformed during their transit through the optical chamber under laminar flow conditions, when they encounter changes in airflow velocity between the inlet and the optical chamber, as previously observed with LOAC2. Therefore, this approach is well suited for atmospheric particle measurements.

Another difficulty associated with observations at small scattering angles is the contribution of stray light contaminations due to scattering of the laser beam inside the optical chamber. Short-term low-intensity fluctuations of the laser may produce variations in stray light that must be removed to avoid confusion with the light scattered by particles crossing the laser beam. A specific computational procedure was developed to subtract this contamination in real time.

Like most OPCs, LOAC detectors (here photodiodes) record the intensity of the light scattered by each particle and identify the maximum of the intensity during the particle transit. The signal recorded as a function of time by the photodiode generally follows a gaussian-like shape [57]. Nevertheless, secondary intensity maxima may occur and can be attributed to the rotation of irregular shaped particles in the airflow [43]. The detection of a new intensity peak is inhibited until the output voltage recorded by the photodiode decreases to a predefined threshold. This procedure prevents from multiple counting of the same particle. The threshold corresponds to the lower limit of the intensity for a given size class. However, such a procedure may lead to an underestimation of particle number concentrations when they are too high, typically of the order of several particles cm^−3^.

### 2.2. LOAC2 Concept and Design

As in the previous version, LOAC2 does not use lenses in front of photodiodes to collect light; therefore, the scattered photons directly illuminate the photodiodes. Consequently, a field of view of few degrees is achievable to record the light, which requires low-noise electronics to accurately detect low scattered intensities. On the other hand, this design prevents optical misalignment issues in the case of vibrations and strong temperature variations, such as those encountered during atmospheric balloon flights, and also avoids fouling problems on the optics.

LOAC2 uses a 650 nm laser diode with an output of 50 mW. The laser beam is homogeneously expanded laterally using a Powell lens to cover the entire optical chamber; therefore, all collected particles cross the laser beam. To minimize the particle transit time and thus increase the detectable concentration limit to several particles per cm^−3^, the beam has a vertical width of about 200 µm, with a gaussian shape profile. The region where the particles cross the beam is slightly out of focus in order to minimize sensitivity to optical misalignment that may occur due to vibrations and variations in temperature and pressure. A light trap and optical baffles are designed to minimize stray light contaminations reaching the photodiodes.

**Figure 1:**
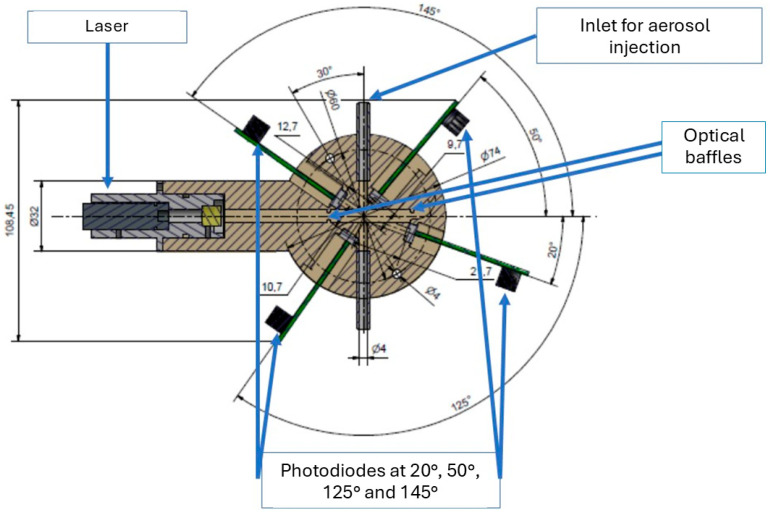
LOAC2 geometry of measurements.

A main innovation of the LOAC2 is that measurements are performed at 4 scattering angles (instead of 2 for the previous version), as shown in Figure 1 of the measurement geometry. This approach is based on the scattering functions of particles, which describe how the intensity of light scattered varies with angle, depending on the particles’ size, refractive index and porosity [58,59]. In general, the darker the particles, the greater the amplitude of the scattering curves at large phase angles. Performing measurements at multiple angles, in addition to the primary one, helps to determine the main optical properties and, consequently, the nature of the particles [60,61,62,63]. The challenge lies in unambiguously identifying the presence of liquid and hydrated particles and distinguishing them from solid particles. The best compromise between instrument weight and size, cost, compact electronics, and the telemetry system for balloon flights is achieved by using 4 scattering angles. Therefore, the 4 angles used are: 20° for counting in the diffraction angle domain, and 50°, 125° and 145° for typology identification.

**Figure 2:**
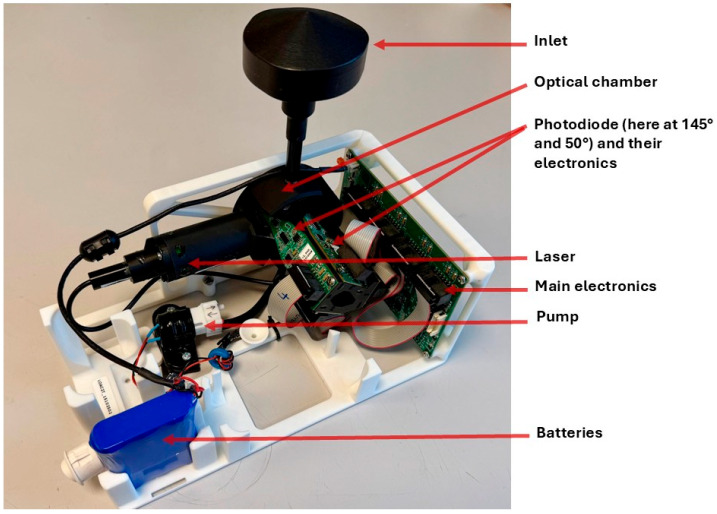
The LOAC2 instrument (mounted on its support for the weather balloon flight gondola).

The main components of the instrument are presented in Figure 2. The pump connected to the optical chamber allows airflow rate of approximately l L.min^−1^. A specific rain cap inlet has been developed to block the possible sunlight contamination as well as rain. The weight of the instrument, including electronics, cables, pump, and inlet is 530 g, with a consumption of 3 W. When used under weather balloons, the total weight of the gondola is about 1 kg, with addition of the batteries, the meteorological probe, and the telemetry system. Raw measurements are obtained every 10 s; concentration and typology data are available every minute in the nominal mode.

LOAC2 can detect particles in the 0.15–50 µm with 19 size classes in the balloon flight version, the size being defined as the optical diameter. For the ground-based version, used for permanent monitoring or field campaigns, the dedicated configuration includes 30 size classes in the 0.15–90 µm range; the additional classes above 10 µm enable the detection and classification of pollens. These differences arise from the fact that the balloon-borne version uses a telemetry system to transmit data in real time, with limited bandwidth. The size classes are given in Table 1.

A “speciation index” (see Section 2.4) is calculated for each of the 3 last channels compared to the first one, as was done for the previous LOAC [43]. The more absorbing the particles, the higher the index and the lower the light scattered. These 3 channels use the same output voltage thresholds (in mV) as those from the calibration of the size classes (Section 2.3), allowing for a direct comparison of counts between the first channel and the others 3. For a given size class and for a given particle concentration recorded in the 20° channel, the signal detected by the other channels decreases as the particle darkness, the porosity or the imaginary part of the refractive index increases. This effect leads to an underestimation of particle’s true size, producing a bias in the size distribution. The speciation index is calculated as the ratio of the true particle size, determined from the first channel, to the biased size for each of the 3 other channels, for all size classes where concentrations are high enough to be statistically significant (see Section 2.4).

### 2.3. Calibration

OPCs often use calibrated spherical solid particles (e.g., [64]). In the case of LOAC2, due to the Mie oscillations, the detected scattered intensity can vary strongly, depending on the exact position of the spherical particles inside the optical chamber. Therefore, instead of detecting a specific scattered intensity for monodisperse spheres, LOAC2 observes a broader intensity distribution. A relative maximum in this distribution represents the most probable scattered intensity, superimposed on contributions from particles that are not located at the center of the chamber.

**Figure 3:**
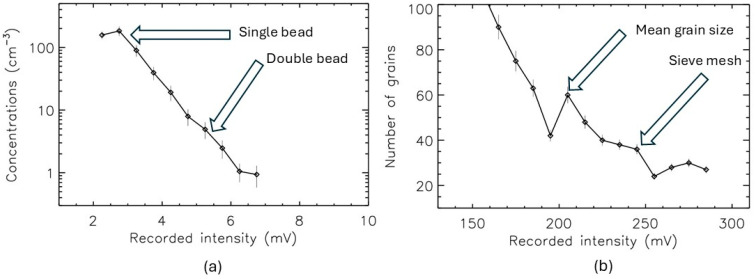
Histogram of the concentration distribution as a function of scattered intensity recorded by the photodiode. (**a**) Latex bead of 300 nm; (**b**) 17 µm silicon carbide irregular grains, using a 25 µm mesh sieve.

For particles smaller than 2 µm, monodisperse latex beads are used. These beads are stored in a liquid suspension, in which remnants of degraded particles and other contaminants may be present, especially when the samples are aged. These contaminants produce a continuum of decreasing concentrations with increasing size, on which the contribution of the beads is superimposed. Also, some bead aggregates may be present, mainly doublets. These aggregates can reasonably be assumed to be non-spherical and therefore do not follow Mie scattering theory; they can be considered as irregular particles for which Mie oscillations at small scattering angle disappear [65]. The optical diameter of such doublets is equal to the diameter of the monomer multiplied by $\sqrt{2}\approx \;$1.4. Figure 3a presents an example of the histogram of the number of detected particles as a function of scattered intensities for 300 nm latex beads. The mean peak for the beads appears at 2.75 mV, while the peak corresponding to doublets appears at 5.25 mV.

**Figure 4:**
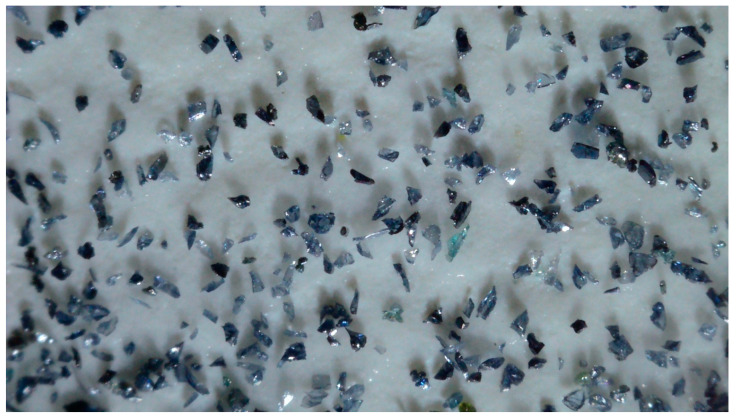
Irregular calibrated silicon carbide grains, with a size distribution centered at 50 µm.

Calibration with solid irregular grains requires a different procedure, in which well-sized calibrated irregular particles are used, with a size distribution following a gaussian profile centered on the most probable grain size. One family of such particles, commonly used for polishing purpose, is silicon carbide (Figure 4), which is easily available from suppliers in broad range of sizes from 5 to hundreds of µm. The main difficulty is that for particles typically smaller than ~30 µm, the grains tend to be agglomerated and must be separated prior to use. A specific procedure has been developed using sieves with square meshes slightly larger than the mean grain size; the sieve is mechanically shaken to select the grains. As a result, the histogram of the number of detected particles as a function of scattering intensities exhibits, in its lower range, the contribution of particles in ambient air, followed by a concentration peak corresponding to the mean grain size, and an upper concentration limit corresponding to largest grains able to pass through the mesh. Finally, grains larger than 40 µm can be directly injected into the optical chamber without sieving. A statistically significant number of grains must be recorded, at least several tens of grains, to reliably retrieve their mean optical properties [66]. Figure 3b presents the concentration histogram for 17 µm silicon carbide. The concentration peak at 210 mV corresponds to the mean particle size, while the concentration drop at 250 mV corresponds to the largest particles able to pass through the 25 µm mesh sieve. Grains detected beyond this size limit are those that can pass by forcing their way through the sieve mesh. This procedure can also be applied to other types of mineral particles. Although such particles are not well-calibrated, roughly size selection can still be achieved using the sieves.

**Figure 5:**
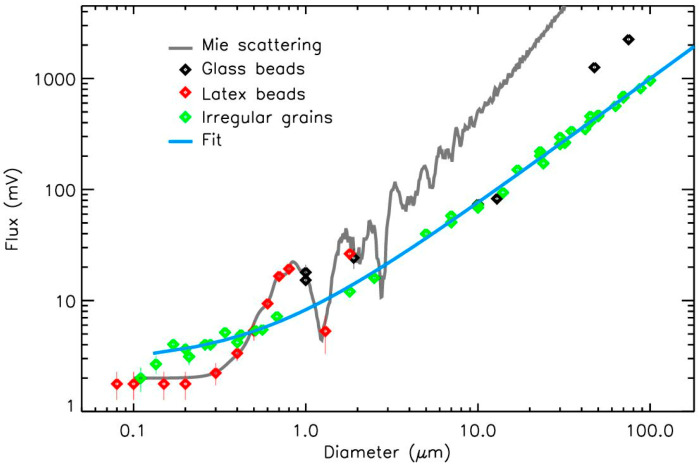
Calibration curve for LOAC2 using different types of particles.

Considering the different types of grains studied, the calibration curve can be established (Figure 5). As expected, latex beads and small glass beads follow Mie scattering calculation. On the other hand, the largest glass beads, with significant surface roughness, can be classified as irregular grains, or as lying in the transition regime between irregular and perfect sphere, depending on their manufacturing quality.

Strictly below 0.3 µm, the scattered light values for perfect spheres are identical, and correspond to the instrumental noise. Between 0.15 µm and 0.4 µm, the values obtained for irregular grains are higher than those for perfect spheres. This could be due to the fact that the transition from the Mie regime to the Rayleigh scattering regime occur for particles lower than about 150 nm, corresponding approximately to λ/4, where λ is the illumination wavelength. For perfect spheres, however, this transition could occur for diameters below ~0.5 µm.

The curve for irregular grains then crosses the Mie oscillations between 0.5 and 3 µm. At larger sizes, the irregular gain curve lies below that of perfect spheres, and the discrepancy increases with particle size. This behavior results from the tube that limits the field of view in front on the photodiodes; it acts as diaphragm, blocking oblique light rays that are not parallel to the tube axis [55]. This effect disappears when the optical field of view is increased to several tens of degrees and therefore does not affect most OPCs. On the other hand, this configuration provides access to a wider size range for a given detector dynamic range.

Since LOAC2 is dedicated to the detection of real atmospheric particles, which are predominantly irregular, the calibration curve is established using fit obtained for the irregular particles (blue curve in Figure 5).

For high concentrations, typically about 100 particles cm^−3^ for particles greater than 1 µm and up to 1000 particles cm^−3^ for particles smaller than 1 µm, the effect of possible overlap during the simultaneous transit of two or more particles through the laser beam becomes non-negligible. In such case, the measured concentrations may be underestimated and should therefore be interpreted with caution.

The errors bars are established to be ± 20% when considering the reproducibility between different copies of the interment, arising from variation in laser output power and the electronics components. The actual counting uncertainty should also include contributions from Poisson counting statistics: 60% for aerosol concentrations of 10^−2^ cm^−3^, 20% for 10^−1^ cm^−3^, and 6% for concentrations higher than 1 cm^−3^.

The electronics have been designed to be insensitive to the electromagnetic radiation encountered both at ground and in the atmosphere. In addition, laboratory tests and in-flight atmospheric measurements have shown that LOAC2 can be operated between −20 °C and 40 °C with unchanged performances. This temperature range refers to the temperature inside the enclosure containing the instrument, while the temperature of the sampled air can be below −70 °C (typically at the tropopause). Furthermore, no degradation of electronic performances occurs in the stratosphere at pressure down to 5 hPa (corresponding to about 35 km, which is the maximum altitude reached by the weather balloons used for the flights). These performances constitute one of the main improvements of the new LOAC2 version compared to the previous one, which was sensitive to temperature and electromagnetic radiation and therefore require corrections.

### 2.4. Typology Determination

The speciation index is calculated using measurements from the last 3 channels compared to those of the first channel. Laboratory measurements, with well-known particles has been conducted to establish reference speciation index curves representing the evolution of the indices with increasing particle size. The particles are grouped into 3 main families: carbon, mineral and water, which are representative of those commonly found in ambient air. The subfamilies are: black carbon, organic carbon, optically absorbent minerals (as concrete dust and granite dust), semi-transparent minerals (such as sands), transparent minerals (such as salt), hydrated solid particles (when the atmospheric humidity is high), water droplets in clouds and fog, sulfuric acid droplets, and ice particles. The particles are injected into the ambient air of a dedicated room for solid and liquid particles, and into the dedicated simulation chamber CESAM for sulfuric droplets [67], at sufficiently high concentrations to make the measurements almost insensitive to the remaining background ambient air particles.

The typology identification is conducted as follows. First, the speciation indices are calculated from ambient air measurements for size classes in which the concentrations measured in the counting channel exceed 10^−2^ cm^−3^. Then, for each size class and for the 3 speciation channels (50°, 125°, and 145°), the distance *D*50, *D*125 and *D*145 are calculated between the measured speciation indices and the reference speciation indices for the 8 species families. To combine the results from the 3 channels and reduce measurement uncertainties associated with each individual channel, the final distance *Df* (an 8-values table) is calculated as follows:

(1) $$Df=\;\sqrt{{\left(D50\right)}^{2}+{\;(D125)}^{2}+{\;(D145)}^{2}}\;$$

The position of the minimum value in the *Df* table is then determined. The corresponding sub-family is finally attributed as the most probable particle type.

In case of low concentrations or strongly optically absorbing particles, no counts may be detected in the third and/or the fourth channel. In such case, the *Df* values are calculated using only the speciation channels and the size classes for which the concentrations are greater than 0. When the concentration in the counting channel is below the 10^−2^ cm^−3^, no identification is possible because the distance calculations become inaccurate.

**Figure 6:**
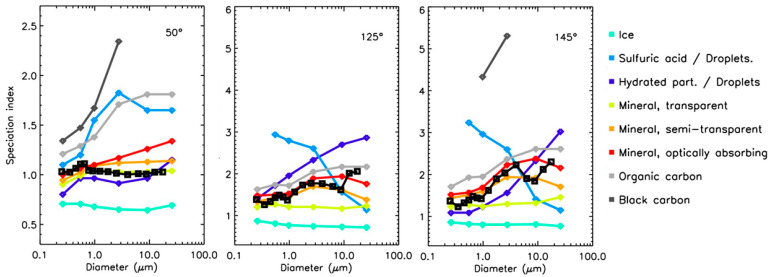
Example of the measured speciation indices for the 3 typology channels, during a balloon flight (14 November 2025 from Orléans, France, 47.8° N–1.9° E) within a layer containing mainly transported Saharan dust at an altitude of 1.3 km. The references values obtained from laboratory measurements are colored curves, while the measurements in the atmosphere are shown in black.

**Figure 7:**
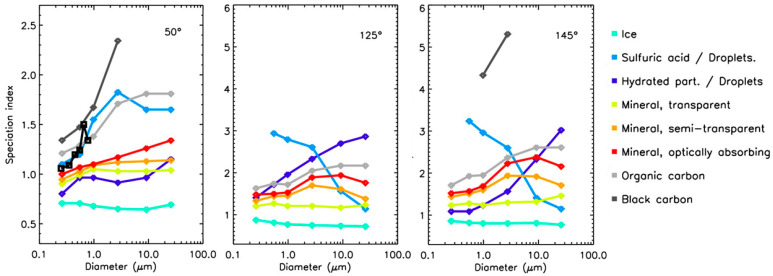
Same as Figure 6, but in the stratosphere at an altitude of 21.2 km, where the typical stratospheric aerosols (sulfuric acid droplets and some carbonaceous particles) are not bright enough to be detectable on the 125° and 145° channels.

For aerosols of a single nature, the identification accuracy for each size class is approximately 90%. Figure 6 presents an example of measured speciation indices during a balloon flight on 14 November 2025 from Orléans, France, within a layer of transported Saharan dust, at an altitude of 1.3 km. Figure 7 presents an example in the stratosphere, where the concentrations are low. The typology retrieval is performed using only the channel at 50° when sulfuric acid or carbonaceous particles are not sufficiently bright to be detected in the 125° and 145° channels.

**Figure 8:**
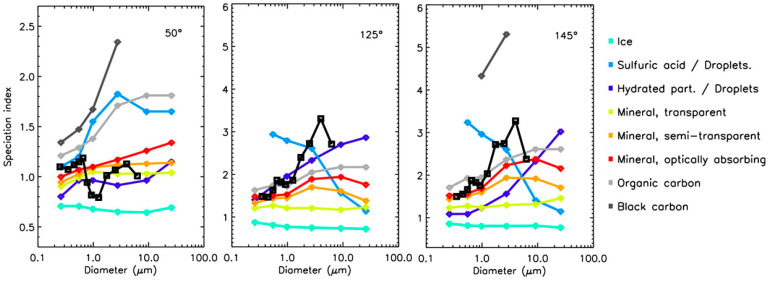
Same as Figure 6, but for mixed background tropospheric aerosols at an altitude of 4.5 km, where no accurate identification can be conducted.

Some uncertainties may arise in the resulting identifications when particles of different natures are simultaneously present, leading to different identifications between adjacent size classes and between consecutive measurements, as presented in Figure 8 for background tropospheric aerosols. In such cases, it is recommended to consider successive temporal identifications and several adjacent size classes to determine the most probable particle type. Nevertheless, when multiple aerosol families coexist and are well mixed in the air, the typology identification may produce inconsistent results between size classes and over time or altitude, and thus should not be considered reliable.

### 2.5. Mass-Concentration Determination

This typology identification is essential for determining mass-concentration from counting measurements. Mass concentrations are generally given for dry particles when considering ambient air quality monitoring [68]. Most solid particles commonly present in ambient air have relatively similar densities, around 2 g·cm^−3^, although some variability has been reported between carbonaceous and minerals particles [69]. The LOAC2 typology identification can help to better determine which density values should be applied. However, when the humidity level is high, typically above 70%, solid particles can be hydrated. Therefore, for a given size, their effective density may decrease. In addition, water droplets may be present under wet atmospheric conditions (e.g., during fog events). This represents one of the main limitations when using OPCs to estimate PM2.5 (particles smaller than 2.5 µm) and PM10 (particles smaller than 10 µm) mass-concentration. Consequently, meteorological parameters must be considered in order to apply appropriate corrections to the measurements [70].

The typology identification makes it possible to distinguish between ice particles, liquid droplets and hydrated solid particles. When the presence of such particles is clearly detected, a density of 0 g·cm−3 can be assumed for micron-sized ice particles and liquid droplets in the context of dry mass-concentration calculations. For highly hydrated submicronic particles containing a solid core, an empirical density of about 0.5 g·cm−3 may be assumed.

## 3. Results

### 3.1. Example of Measurements

**Figure 9:**
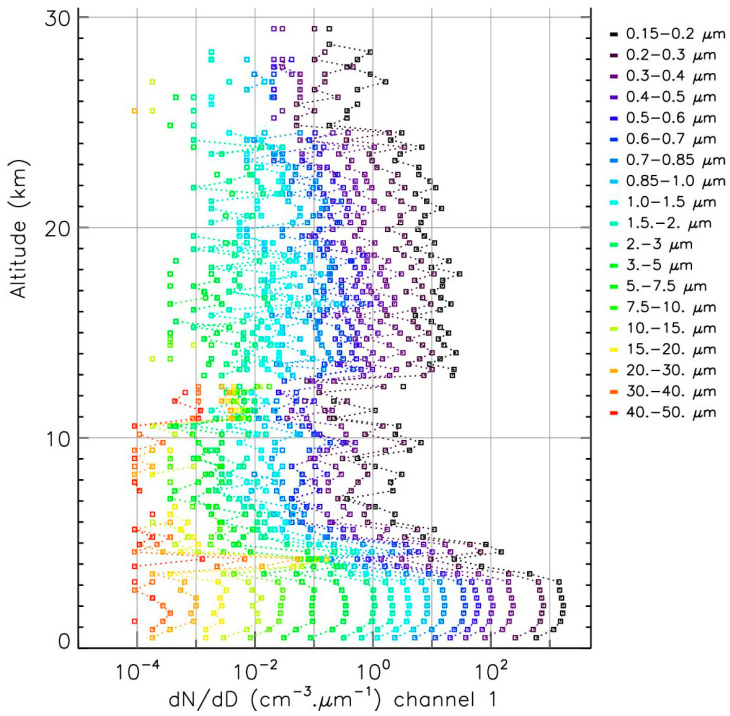
Vertical profile of number concentrations for the 19 size classes during the LOAC2 weather balloon flight on 14 November 2025, launched from Orléans (France, 47.8° N–1.9° E). No smoothing has been applied on the profile. Channel 1 corresponds to the first angle measurements dedicated to particle counting.

**Figure 10:**
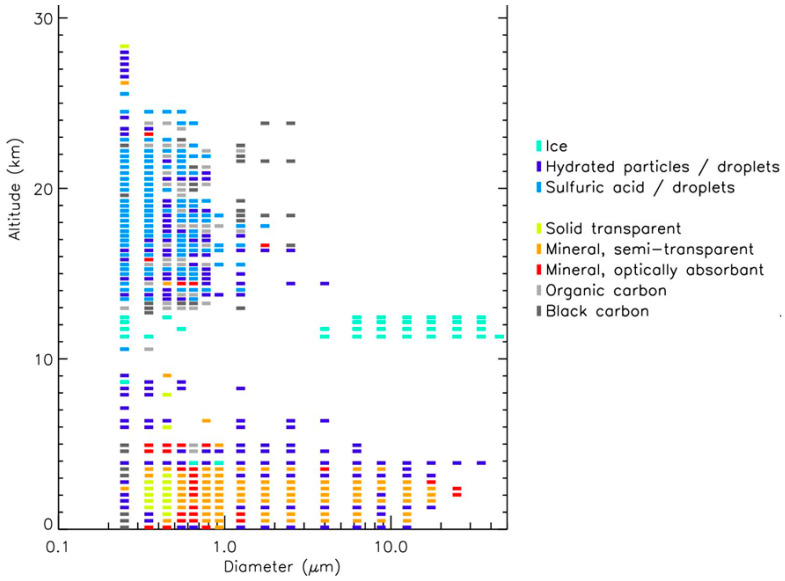
Vertical profile of the typologies for the 19 size classes during the LOAC2 weather balloon flight on 14 November 2025 launched from Orléans (France, 47.8° N–1.9° E).

LOAC2 is an instrument designed to operate outdoors during field campaigns with minimal maintenance and it can be used several times for balloon flights up to the middle stratosphere. As an example, the LOAC2 weather balloon flight conducted on 14 November 2025, for which the typology example is presented in Figure 5, illustrates the instrument’s performances and the different types of particles that can be encountered. The measurements were conducted during balloon ascent at a mean speed of ~5 m·s^−1^, and the vertical profile representing the evolution of number concentration (dN/dD corresponds to the number concentration in a size lass divided by the width of that size class) and particle typology with altitude are presented in Figure 9 and Figure 10.

The concentration and typology profiles show a transported Saharan dust layer extending from the ground up to an altitude of 5 km, in agreement with previous balloon-borne measurements during this kind of event [45]. Cirrus clouds, characterized by ice particles larger than 10 µm, are then clearly detected between 11 and 13 km. Finally, above 13 km, the stratospheric aerosol layer shows a maximum concentration below 20 km, followed by a decrease at higher altitude. The typology mainly indicates sulfuric acid droplets, although a small signature of hydrated solid particles, water droplets and carbonaceous materials may also be present, as previously detected in the stratosphere [39,71]. The origin of these particles may be linked to a remaining signature of smoke emitted from large wildfires, likely internally mixed with sulfuric acid droplets, which can inject such particles up to the middle stratosphere [72].

### 3.2. Intercomparison with Other Instruments

It is necessary to assess the performance of LOAC2 and to compare its measurements with those from other instruments, although no absolute reference instrument exists for aerosol counters. As a result, this exercise is more an intercomparison than a validation. Their measurements depend on the type of particles used for calibration and on the definition of particle diameter that is adopted [73].

**Figure 11:**
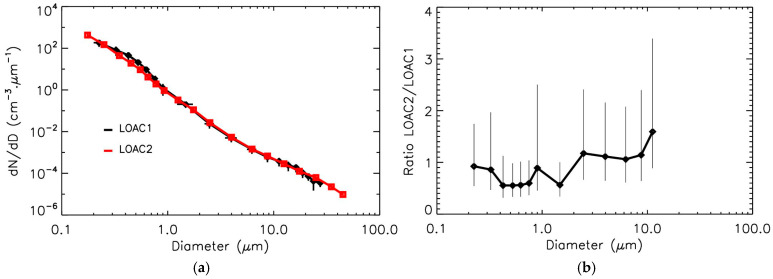
Comparison of the granulometries obtained with LOAC1 and LOAC2 under indoor air conditions with CaCO_3_ particles in suspension; (**a**): size distribution; (**b**): ratio of the concentrations for particles smaller than 10 µm.

The first intercomparison session was conducted with the previous LOAC version (here referred to as LOAC1), under indoor air conditions with CaCO_3_ particles in suspension (Figure 11a). The granulometry results (particle size versus concentration) are visually in good agreement, considering the error bars. To estimate the level of agreement, the LOAC2 measurements were linearly interpolated onto the same size range as LOAC1, although such procedure may introduce uncertainties because particle concentrations generally follow lognormal or power law distribution as a function of particle size. The LOAC2/LOAC1 ratio is presented in Figure 11b for particles up to 10 µm (results for larger particles being instable because of the low concentration and the interpolation procedure). The ratio remains relatively close to unity, with a mean value of 0.9 ± 0.6. An analysis of the temporal evolution for the measurements for submicronic particles confirms that the LOAC2 measurements are more stable over time than those of LOAC1.

The second intercomparison session was conducted in the CESAM atmospheric simulation chamber [74] using ambient air, ammonium sulfate, and sulfuric acid particles. Two other aerosol counters were available for comparison with LOAC2: a Grimm OPC providing counting in 30 size classes between 0.265 and 31 µm, and a DMPS providing counting in 45 size classes from 5 to 800 nm. Such intercomparison sessions are not straightforward because the different instruments do not use the same size classes. In addition, the DMPS and Grimm OPC do not provide errors bars or information on the measurements’ uncertainties, which may depend on the aerosol nature. Nevertheless, to provide an estimate of the LOAC2 performance, the LOAC2 measurement were interpolated onto the size range of the other instruments. LOAC2 was installed inside the chamber, whereas the two other counters were located outside and sampled the particle inside the chamber through a pipe, in which sedimentation may occur for the largest particles [75].

**Figure 12:**
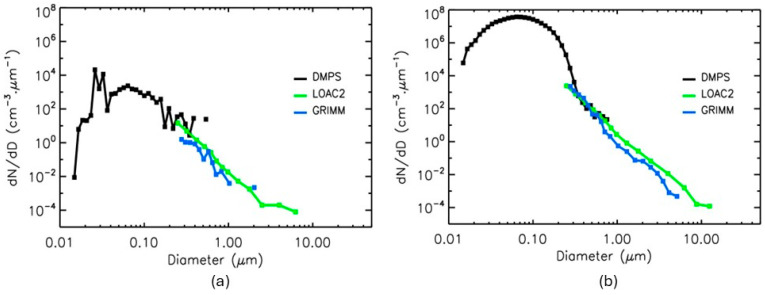
Comparison of granulometries measured by LOAC2, Grimm, and DMPS in the CESAM atmospheric chamber. (**a**) Ambient air conditions with low concentrations. (**b**) Ammonium sulfate aerosols.

Table 2 presents the considered size range and the mean ratio of LOAC2 concentrations relative to those measured by the two other instruments, for both ambient air and ammonium sulfate aerosols shown in Figure 12. The agreement with the DMPS is good for submicronic particle concentrations within the common size range. The Grimm and LOAC2 measurements show only moderate agreement in absolute values but follow the same overall trend (Figure 12). The differences may result from discrepancies in size calibration, but also from sedimentation effect affecting for the aerosols sampled by the Grimm instrument.

**Figure 13:**
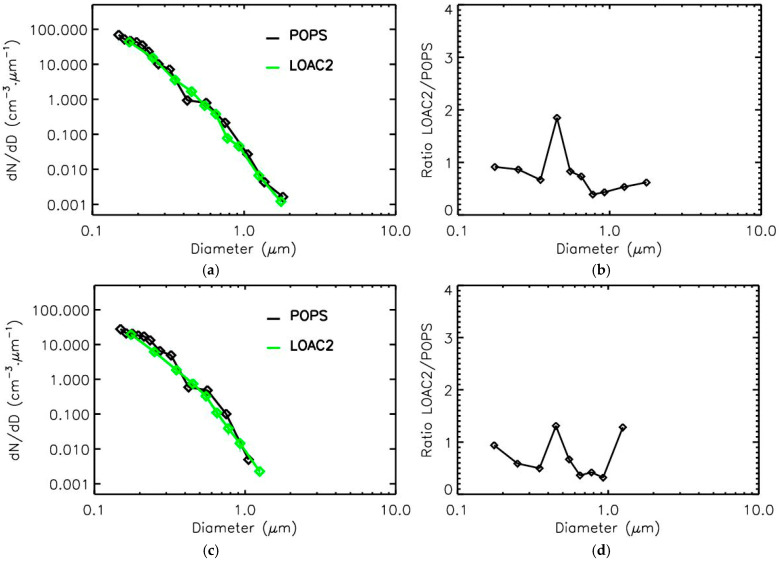
Comparison of the granulometries measured by LOAC2 during ascent with POPS measurements in the same gondola. The flight was conducted on 17 December 2025 from Orléans, France. (**a**,**b**): concentrations and ratios at an altitude of 16.5 km; (**c**,**d**): concentration sand ratios at an altitude of 19.5 km.

The third intercomparison session was conducted during a weather balloon flight. LOAC2 measurements obtained during the balloon ascent are compared to those of the balloon-borne POPS OPC [51], mounted in the same gondola as LOAC2, during a flight on 17 December 2025 from Orléans, France. Since POPS is calibrated for sulfuric acid/water droplets, only measurements within the stratosphere where such particles are dominants (mainly liquid sulfuric acid and water droplets, with expected mass percentages of about 75% and 25% respectively) are considered. The POPS measurements were interpolated onto the same size range as LOAC2. Figure 13 presents the granulometries measured by the two instruments and their corresponding ratios. The agreement is again satisfactory, considering the different calibration methods, with both instruments showing the same decrease in concentrations with increasing particle size. The ratios of LOAC2/POPS are equal to 0.8 ± 0.4 and 0.7 ± 0.4.

Although the LOAC2 measurements were slightly higher than those of the Grimm instrument during the previous intercomparison session, they are slightly below those of the POPS instrument for this session. Considering also the good agreement with the DMPS instrument, no systematic underestimation or overestimation of LOAC2 concentrations relative to the other counting instrument could be identified.

The last intercomparison session concerns indirect measurements, in which LOAC2 particle counts are converted to mass concentrations of dry PM10 and PM2.5 and compared to measurements of legally regulated air quality monitoring stations. These stations use a gravitational technique, or an equivalent method, to determine the cumulative mass of all particles smaller than 10 µm and 2.5 µm. However, these measurements can be subject to uncertainties of several µg·m^−3^ [76,77].

Almost one year of ground-based measurements were conducted at two locations in France: one in the campus (with lots of vegetation) of the LPC2E-CNRS laboratory in Orléans, next to a reference station (47.8377° N, 1.9446° E), and one in Paris, at the André Citroën park, on the gondola of the touristic balloon Generali (48.8413° N, 2.2739° E) [46], located 5 km from a reference background urban station in central Paris. Such intercomparisons are quite challenging due to the wet and windy weather in northern France during that year, which prevented the occurrence of high pollution events. The LOAC2 typology is dominated by droplets, hydrated solid particles and mineral particles at the LPC2E-CNRS park, whereas submicron carbonaceous particles and micron-sized minerals particles are predominant at Parc André Citroën park.

**Figure 14:**
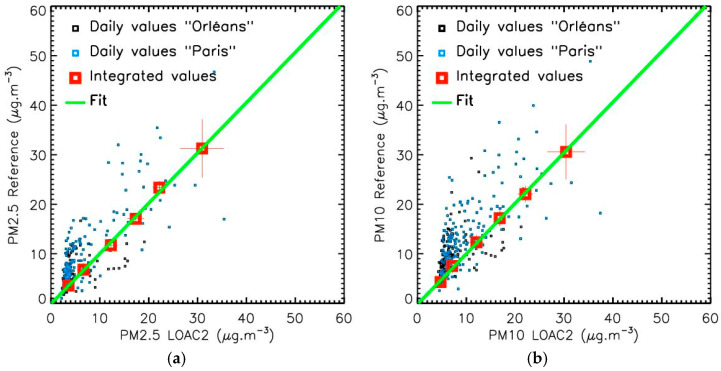
Scatter plots comparing LOAC2 and reference mass concentrations (from Orléans and Paris measurements) The daily values are integrated by steps of 5 µg·m^−3^ for daily values below 25 µg·m^−3^, and in a bin of 25 µg·m^−3^ above this threshold, in order to improve statistical significance. (**a**) PM2.5. (**b**) PM10.

All the data are averaged daily to match the reference values provided by the air quality stations. Because of low pollution levels in 2025 and values close to the uncertainties, the measurements from the two locations are merged. As shown in Figure 14, the Orléans and Paris datasets exhibits similar distribution, which justifies combining them. Since the mass concentrations are not regularly distributed, the daily values are integrated in bins of 5 µg·m^−3^ for daily values below 25 µg·m^−3^, and in a bin of 25 µg·m^−3^ above this threshold, in order to improve statistical significance. Then, the correlation between LOAC2 and the reference stations is very good (Table 3), demonstrating LOAC2′s ability to remove the contribution of droplets. Otherwise, due to their concentrations, the calculated mass concentrations could reach values of several 100 µg·m^−3^, assuming the density of solid particles. However, this needs to be confirmed for higher pollution levels.

### 3.3. Flights Under Alert

One advantage of such a lightweight instrument is its ability to perform weather balloon flights under short notice, in order to study specific atmospheric events such as plumes from major wildfires, volcanic eruptions, or meteoritic disintegration. Obviously, such flights are conducted when meteorological conditions permit, with no rain and ground-level speeds lower than about 15 m·s^−1^.

As an example, this strategy was successfully implemented on 3 June 2025 from Orléans, France, during the passage of a wildfire plume originated from Canada. The presence of the plume above Orléans has been confirmed the previous day using the Atlid/EarthCARE instrument [78] (measurements maps were notably available at aerosolstrato.projet.latmos.ipsl.fr). Forecast trajectories have been calculated using Hysplit model (https://www.ready.noaa.gov/HYSPLIT.php, accessed on 1 June 2025) to ensure interception of the plume and to adjust the launch time.

**Figure 15:**
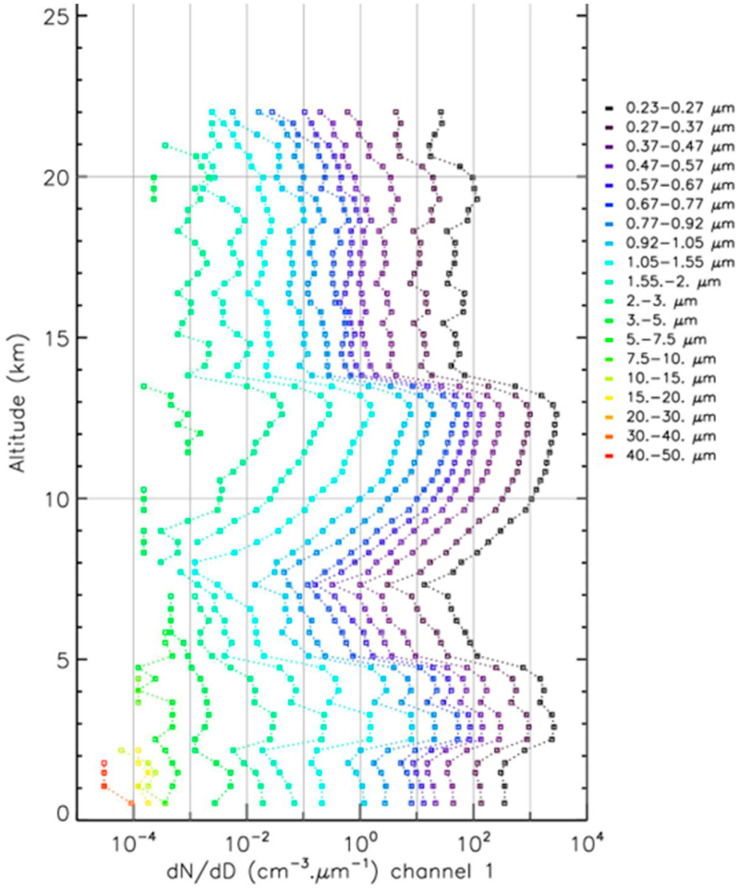
Vertical profile of number concentrations for the 19 size classes during the LOAC2 weather balloon flight on 3 June 2025, launched from Orléans (France, 47.8° N–1.9° E), with a 3-point moving average for each size class. Note that the size classes differ from the nominal configuration, due to a post-flight recalibration.

The vertical profile (Figure 15) shows the wildfire plume extending from 8 to 13.5 km, with a sharp upper boundary, encompassing the tropopause, which was located at 11.5 km at that time. This indicates that part of the plume had entered the stratosphere, likely through the pyroconvection process [79]. The maximum concentration within the plume is about 100 times higher than in the surrounding layers, with values comparable to those observed at ground level during urban pollution events in cities such as Paris, France [46].

These in situ measurements are important for estimating particle size distribution and concentrations, and thus for better constraining modeling studies on long-range plume transport and their implications for local atmospheric chemistry as well as the radiative balance.

## 4. Conclusions

The new Light Optical Aerosols Counter LOAC2 provides number and mass concentrations of liquid and solid aerosols from 0.15 µm to 50 or 90 µm (depending on its configuration), as well as an estimate of the main particle typologies. It can be used outdoor for field campaigns and monitoring with little maintenance, and can also operate during balloon flights under harsh atmospheric conditions.

The intercomparison sessions conducted in 2025 under different conditions have shown that LOAC2 is in good agreement with other instruments, giving confidence on the LOAC2′s ability to detect aerosols in different atmospheric environments, even in low concentrations, and to identify their typology. Nevertheless, there is a risk of underestimation when concentrations exceed 100 particles cm^−3^ for particles greater than 1 µm and 1000 particles cm^−3^ for particles smaller than 1 µm, conditions that may typically be encountered in soot plumes close to the emission sources. LOAC2 is now routinely used during weather balloon flights. One main project for the 2025–2026 period is the validation of the extinction vertical profiles obtained with the Atlid instrument onboard the ESA/JAXA EarthCARE satellite [78] through conversion of the size distributions to extinction using a Mie scattering model. Seventy flights are conducted mainly from France. In addition, flights can easily be conducted worldwide during specific events, as Saharan dust plume, and wildfires and volcanic plume associated with high concentrations of aerosols in the stratosphere.

LOAC2 is also used on the touristic balloon Generali in Paris to monitor pollution levels from ground up to an altitude of 300 m, continuing the measurements performed since 2013 with the previous version of LOAC [46].

Finally, a laboratory database for pollens will be established in the following month, in order to unambiguously identify pollens and their main family in the LOAC2 measurements. This approach will progressively allow the replacement of the pollen measurements previously conducted with the Beenose aerosol counter [19].

LOAC2 will then become a unique instrument with a counting capability enabling the measurements of anthropogenic pollution, natural mineral particles, and bioaerosols, as well as particle typology identification. It can be used both at ground and in the atmosphere up to about an altitude of 35 km, depending on the scientific objectives of the users.

## Figures and Tables

**Table 1:** LOAC2 size classes.

Balloon Version (Using Telemetry)	Ground-Based Version
0.15–0.2 0.2–0.3 0.3–0.4 0.4–0.5 0.5–0.6 0.6–0.7 0.7–0.85 0.85–1.0 1.0–1.5 1.5–2.0 2.0–3.0 3.0–5.0 5.0–7.5 7.5–10.0 10.0–15.0 15.0–20.0 20.0–30.0 30.0–40.0 40.0–50.0	0.15–0.2 0.2–0.3 0.3–0.4 0.4–0.5 0.5–0.6 0.6–0.7 0.7–0.85 0.85–1.0 1.0–1.5 1.5–2.0 2.0–3.0 3.0–5.0 5.0–7.5 7.5–10.0 10.0–12.5 12.5–15.0 15.0–17.5 17.5–20.0 20.0–22.5 22.5–25.0 25.0–27.5 27.5–30.0 30.0–35.0 30.0–40.0 40.0–45.0 45.0–50.0 50.0–60.0 60.0–70.0 70.0–80.0 80.0–90.0

**Table 2:** Mean value of the ratios between LOAC2 and the DMPS and the Grimm instruments.

Type of Particles	LOAC2/DMPS 0.15–0.76 µm Range	LOAC2/Grimm 0.28–0.72 Range
Ambient air	1.4 ± 0.4	8.1 ± 1.4
Ammonium sulfate	1.4 ± 0.4	2.7 ± 0.6

**Table 3:** Statistics of the fits in Figure 14.

	Slope	Value at the Origin	Correlation
PM2.5	1.02 ± 0.16	−0.12 ± 1.97	0.99
PM10	1.0.2 ± 0.18	−0.23 ± 2.27	0.99

## Data Availability

Dataset available on request from the authors.
